# The Potential of a Digital Weight Management Program to Support Specialist Weight Management Services in the UK National Health Service: Retrospective Analysis

**DOI:** 10.2196/52987

**Published:** 2024-01-24

**Authors:** Rebecca Richards, Gina Wren, Michael Whitman

**Affiliations:** 1 Second Nature London United Kingdom; 2 Nuffield Department of Primary Health Care Sciences University of Oxford Oxford United Kingdom

**Keywords:** digital health intervention, smartphone, diabetes management, obesity management, mobile phone, management, obesity, digital health, diabetes, weight, manage, support, weight management, retrospective analysis, treatment, type 2 diabetes, effectiveness, primary care, weight loss, clinical, primary care service

## Abstract

**Background:**

Digital weight management interventions (DWMIs) have the potential to support existing specialist weight management services (SWMS) in the National Health Service (NHS) to increase access to treatment for people living with obesity and type 2 diabetes. At present, there is limited real-world evidence and long-term outcomes on the potential effectiveness of DWMIs to support such services.

**Objective:**

This study aimed to examine real-world data to evaluate the impact of Second Nature’s 12-month DWMI for patients living with obesity with or without type 2 diabetes, referred from NHS primary care services, on sustained weight loss over a 2-year period.

**Methods:**

Retrospective data were extracted in August 2023 for participants who participated in the program between January 1, 2017, and January 8, 2021. Eligible participants were adults with a BMI ≥35 kg/m^2^, with or without type 2 diabetes. The primary outcomes were weight change in kilograms and percentage weight change at 2 years. Secondary outcomes were weight loss at 1 year, program engagement, and the proportion of participants who achieved ≥5% and ≥10% weight loss. Differences in weight loss between baseline and the 1- and 2-year follow-up points were compared using paired 2-tailed *t* tests. Linear regression models were used to examine whether participants’ ethnicity, indices of multiple deprivation, presence of type 2 diabetes, or program engagement were associated with weight loss at 1 year or 2 years.

**Results:**

A total of 1130 participants with a mean baseline BMI of 46.3 (SD 31.6) kg/m^2^ were included in the analysis. Of these participants, 65% (740/1130) were female (mean age 49.9, SD 12.0 years), 18.1% (205/339) were from Black, Asian, mixed, or other ethnicities, and 78.2% (884/1130) had type 2 diabetes. A total of 281 (24.9%) participants recorded weight readings at 2 years from baseline, with a mean weight loss of 13.8 kg (SD 14.2 kg; *P*<.001) or 11.8% (SD 10.9%; *P*<.001). A total of 204 (18.1%) participants achieved ≥5% weight loss, and 130 (11.5%) participants reached ≥10% weight loss. Weight loss did not significantly differ by ethnicity, indices of multiple deprivation, presence of type 2 diabetes, or engagement in the program.

**Conclusions:**

The findings suggested that Second Nature’s DWMI has the potential to support people living with obesity and type 2 diabetes remotely to achieve clinically significant and sustained weight loss at 2 years from baseline. Further research is needed to compare the intervention to standard care and assess integration with multidisciplinary clinical teams and pharmacotherapy in order to support this study’s findings.

## Introduction

### Background

Most adults in the United Kingdom (UK) (around 65%) are affected by overweight or obesity, with the prevalence continuing to rise [1-4]. Due to the complex and chronic nature of obesity and its associated conditions, such as type 2 diabetes [5-7], the annual cost to UK society is estimated to be US $68.6 billion, roughly equivalent to 2-3% of gross domestic product [8].

Treatment for overweight and obesity in the UK broadly consists of 4 tiers of weight management service [9]. Tier 1 includes population-wide, universal, prevention interventions that reinforce messages of healthy eating and physical activity. Tier 2 includes community-setting lifestyle interventions delivered by a health coach, sometimes as part of a multicomponent weight management service, which may include pharmacotherapy. Tiers 3 and 4 are described as “specialist weight management services” (SWMSs) for people living with obesity, and they provide specialist assessment, monitoring, and comprehensive tailored treatment by a clinician-led, multidisciplinary team (MDT). An MDT typically includes a doctor, nurse, dietitian, psychologist, and a physiotherapist or exercise therapist, each with a specialist interest in obesity. Treatment in tier 3 may include pharmacotherapy and support from a dietitian, psychologist, and physiotherapist or exercise therapist where required. Treatment in tier 4 includes preoperative assessment for, and delivery of, bariatric surgery, further supported by an MDT.

While evidence for SWMSs in the UK is limited, short-term data suggest that they can be an effective obesity treatment [10]. For example, a systematic review of 19 studies of SWMSs in the UK reported positive effects on weight (specifically, 43.4% and 29.4% achieved ≥5% and 10% weight loss, respectively), BMI, glycemic control, blood pressure, and physical activity at 12 months [10]. While treatment duration varies between 6 and 24 months, to our knowledge, there are no published data on long-term outcomes following discharge from SWMSs [10,11].

Unlike tier 2, the provision of, and access to, SWMSs across the UK remains limited and varies geographically due to a lack of funding [12]. Similarly, due to the high costs associated with delivering these specialist services, existing services face increasing problems such as long waiting lists, understaffing, and a lack of treatment flexibility, and therefore, treatment often varies between services [11-13]. These barriers can result in treatment delays and adversely affect patient outcomes [11]. As a result, in June 2023, a US $50.9 million 2-year pilot program was announced by the UK government that aims to increase access to newly approved weight loss medication, semaglutide, outside of hospital settings, by using commercial digital weight management providers [14,15]. Furthermore, in August 2023, the National Institute for Health and Care Excellence announced an early value assessment of semaglutide treatment provided by commercial digital weight management providers [16].

Digital weight management interventions (DWMIs) offer a promising addition or alternative to traditional SWMSs that historically have been provided in person [10,17,18]. Potential benefits of DWMIs include increased access to services for some people, increased convenience, more frequent care, resource- and cost-savings, and the potential scalability to help manage the increasing prevalence of obesity and related conditions [16,18]. Previous systematic reviews have shown that DWMIs can be as effective as in-person interventions for weight loss and related outcomes for people with obesity [19-21], and the COVID-19 pandemic provided further evidence that existing intensive, in-person programs could be effectively transformed to deliver care remotely and effectively using technology [22-24]. Furthermore, 2 studies have shown that remote delivery of a weight management program in the UK can be as effective as usual face-to-face support in a tier 3 weight management service [18,25]. For example, a dietetic weight loss app program was found to be as effective and feasible when delivered remotely from a hospital-based SWMS to their usual face-to-face care [25]. However, real-world evidence of the potential for digital intervention to support SWMS in the UK National Health Service (NHS) remains limited [26].

### This Study

To build on this growing evidence base, this study aimed to explore the potential of Second Nature’s [27] DWMI to expand SWMSs outside of hospital settings for NHS-referred patients. It also aimed to contribute real-world evidence of DWMIs and longer-term outcomes following discharge from a weight management service. This retrospective analysis examined real-world data for patients living with obesity with or without type 2 diabetes, referred from NHS primary care services. The impact of Second Nature’s 12-month program on weight change at 2 years from baseline was evaluated. This program was delivered via a smartphone or web-based app and has been found to be an effective weight management intervention and diabetes-related weight management intervention for patients with overweight, obesity, and type 2 diabetes referred by the NHS [28,29]. Previous research has found that DWMIs typically require a high amount of personal agency to be effective, given that making such changes to health behaviors requires time, resources, and education [30,31]. Consequently, such interventions risk exacerbating health inequalities and may be inequitable [30,31]. For this reason, this study also examined whether weight loss differs by ethnicity, socioeconomic status, type 2 diabetes status, and program engagement.

## Methods

### Ethical Considerations

This study did not require institutional review board approval, as it was a service evaluation and did not include personally identifiable information. As per General Data Protection Regulations, participants could request to have their information deleted at any time.

### Participants

For participants who met our eligibility criteria, retrospective data were extracted directly from Second Nature’s database in November 2023, deidentified, and pseudonymized using identification numbers. To be referred to the Second Nature program, participants were required to consent for their anonymized data to be collected for research purposes, including analysis and publication. When registering for the program, participants were asked to agree to a privacy policy that reminded them of their consent. Participants included in this analysis participated in the Second Nature weight management program between January 1, 2017, and January 8, 2021. No major changes were made to the program content during this time.

**Figure 1:**
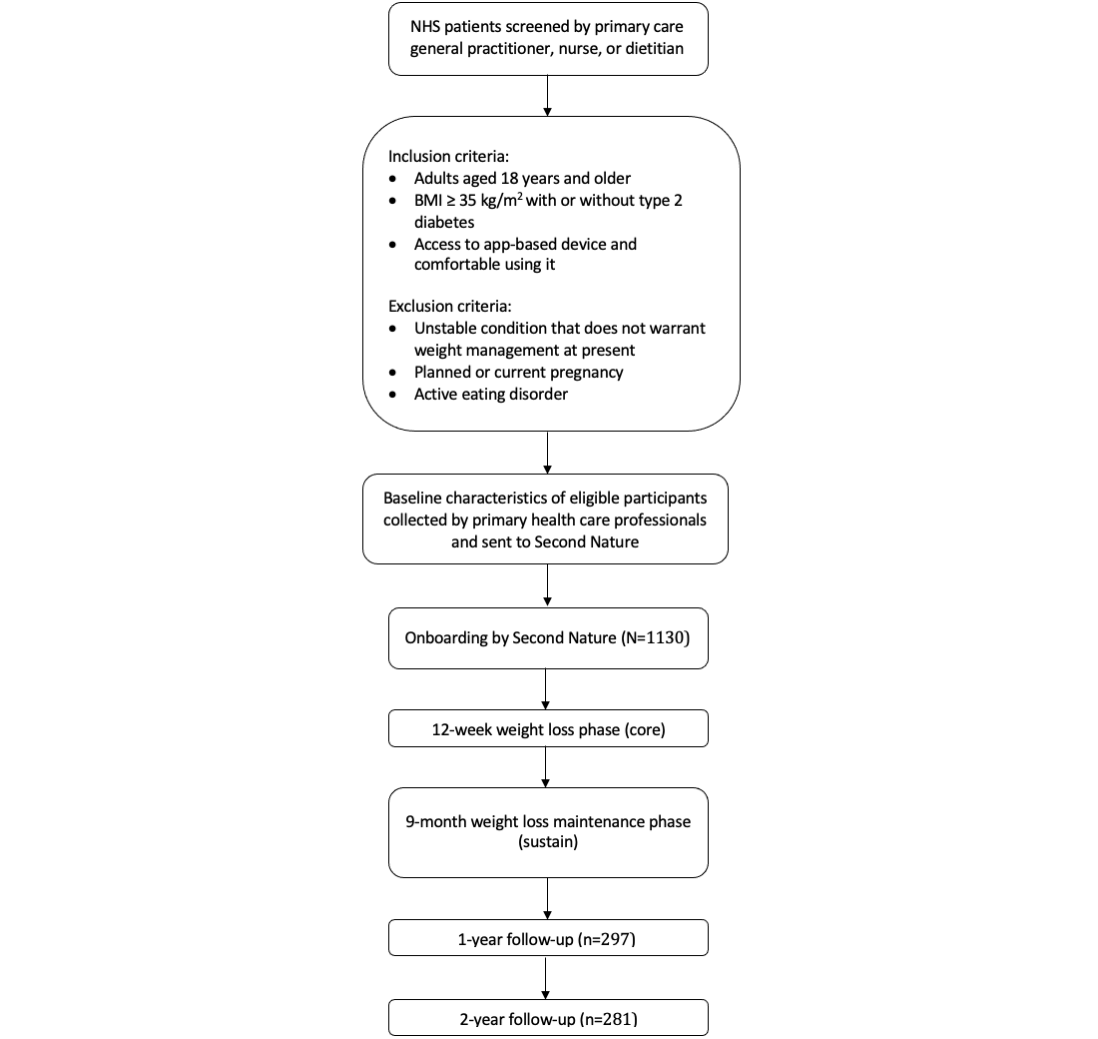
Participant flowchart. NHS: National Health Service.

Participants included in this analysis were screened and referred via secure NHS email to Second Nature by their NHS primary care general practitioner, nurse, or dietitian for weight management support (plus structured diabetes education for participants with obesity and type 2 diabetes). Eligible participants were adults (aged 18 years and older) with a BMI ≥35 kg/m^2^, with or without type 2 diabetes. Participants were required to have access to a smartphone or tablet device and to be comfortable using technology to participate in the Second Nature program. Participants were referred to Second Nature if they were deemed clinically suitable for the program by the referrer, in relation to our inclusion and exclusion criteria. Exclusion criteria included an unstable condition that does not warrant weight management at present, planned or current pregnancy, and an active diagnosis of an eating disorder. Figure 1 presents the participant flowchart.

### Intervention Description

Second Nature’s digital weight management program is a 12-month program, accessed by smartphone or web-based app, and consists of 2 phases: an initial 12-week phase that focuses on weight loss (called “core”) followed by 9 months focusing on maintenance of weight loss (called “sustain”). Participants were encouraged to engage with this program for at least 12 months; however, they retained access to the program and resources indefinitely. The program is available in 10 different languages.

Prior to starting the program, each participant received a recipe book, an instructional handbook, and wireless weighing scales. Throughout each of the phases, participants were given access to educational material on a variety of health and wellness topics such as nutrition guidelines, increasing physical activity, stress management, and improving mental well-being. Participants with type 2 diabetes also received additional structured education modules on managing their condition (accredited by an independent body, Quality Institute for Self Management Education and Training), including the role of insulin and managing their nutritional needs. The program was developed by an MDT of medical doctors, psychologists, dietitians, nutritionists, and behavioral scientists in line with relevant National Institute for Health and Care Excellence guidance for obesity and type 2 diabetes management and behavior change [32-37]. Behavior change techniques and insights were also adopted from the NHS Diabetes Prevention Programme guidelines [38] and the “behaviour change wheel” [39], with new behaviors encouraged through self-monitoring, goal setting, social rewards, and education from credible sources.

Features of the program include daily educational papers and goals; weight, steps, and sleep tracker; and a toolbox of resources (educational materials, recipes and meal planner, journal and food diary, and guided exercise videos). Each participant is assigned a health coach, who provides one-to-one tailored guidance through private text-based communication available during normal working hours, Monday to Friday. Additionally, participants had access to a group chat feature for peer support. The group chat was supervised by a health coach. Engagement with the app was monitored automatically, and health coaches were alerted when a participant showed low engagement (defined as <10 interactions) to indicate the risk of disengaging. Alerts prompted coaches to provide additional support for these participants in the form of messages. Support from their health coach ended following the completion of the 12-week “core” weight loss phase. Health coaches were dietitians (registered with the Health and Care Professions Council) or nutritionists (registered with the Association for Nutrition). Where a participant was coached by a nutritionist, supervision was provided by a dietitian.

Second Nature’s health coaches and participants’ primary care team communicated when necessary throughout the program to ensure safe, effective, and joined-up care. Communication took place through ad hoc phone calls and secure NHS email exchanges. Health information was shared when relevant to discuss and review participants’ progress and challenges. Using this MDT approach ensured continuous monitoring of clinical measures and adjustments to medications, where needed. For example, if participants with type 2 diabetes were using a hypoglycemia-inducing medication, medication was adjusted based on weight loss progress.

### Data Collection

Baseline characteristics (weight, height, age, gender, type 2 diabetes diagnosis, and ethnicity) and contact details were collected by the participant’s primary care referrer and emailed to Second Nature. These data were entered into Second Nature’s referral management system, and participants were sent an email link to complete a series of onboarding questions about their mobility, physical barriers to exercise, motivation, eating behaviors, and diabetes medication. Postcode data were also collected during onboarding to calculate socioeconomic deprivation based on the index of multiple deprivation (IMD) [40].

Participants were sent wireless weighing scales so that they could transfer their weight data to Second Nature. Instructions accompanying the scales advised placement on a firm, flat surface, weighing first thing in the morning after using the restroom, and on the same day at the same time each week to ensure accurate and consistent measurements. After use, the scales automatically transmitted readings to Second Nature’s central database. A weight validation algorithm was used to ensure accuracy, accepting only measurements within a predicted range, considering the last recorded weight and the time since. Any irregular readings prompted an email alert to the participant to explain the reading would not be saved; however, if this was a mistake, then participants could contact their health coach or email the support team. This method aimed to filter out anomalous readings (such as readings from another member of a household), ensuring reliable data for analysis.

Weight readings at baseline, 1 year, and 2 years from the participant’s start date of the program were extracted for the database. The lowest valid weight reading and the closest reading, after 1 year and 2 years, were used for analysis.

Engagement data were continuously collected as users engaged with the program and stored in Second Nature’s secure analytics database. Engagement was defined as the total number of interactions with the app or web-based platforms and only analyzed during the first 3 months of the “core” active intervention phase of the program. Activity was only monitored during this active intervention phase as the intensity of the intervention decreased after 12 weeks, and participation was encouraged less frequently during the maintenance phase.

### Statistical Analysis

The primary outcomes were weight change in kilograms and percentage weight change at 2 years. Secondary outcomes were weight loss after 1 year, program engagement, and the proportion of participants who achieved ≥5% and ≥10% weight loss.

Descriptive statistics were used to examine baseline characteristics of the study population, weight loss (percentage and kilograms), and engagement with the program. Continuous values are presented as mean (SD), and categorical data as n (%), unless otherwise stated.

For the primary analysis, differences in weight between the baseline and the 1- and 2-year follow-up points were compared using paired 2-tailed *t* tests. For each observation, we only compared those with available weight readings at each time point. Data were also analyzed on an intention-to-treat basis, using the baseline weight observation carried forward (BOCF) method when a final weight was not available [41] and using completers only (ie, participants with complete data at all time points), to confirm the validity of the findings and illustrate the pattern of weight change in the same individuals over time.

A series of linear regression models were used to examine the association between baseline characteristics (ethnicity, IMDs, and presence of type 2 diabetes) and weight loss at 1 year and 2 years. Each characteristic was added as an independent variable into separate models to test for factors independently associated with weight loss. In each model, weight loss at either 1 year or 2 years was the dependent variable, and baseline weight was included as a covariate.

A further linear regression model was used to examine the association between program engagement and weight loss at 1 year and 2 years. Engagement was included as the independent variable, the dependent variable was weight loss, and baseline weight was included as a covariate.

All statistical analyses were performed using the R open-source statistical language through the RStudio interface (R Foundation for Statistical Computing), and the criterion for statistical significance was *P*<.05.

## Results

### Baseline Characteristics

A total of 1130 participants were included in this analysis. Of these participants, 740 (65%) were female. The mean age was 49.9 (SD 12.0) years, and the mean baseline BMI was 46.3 (SD 31.6) kg/m^2^. In total, 78.2% (n=884) of participants included in the sample had type 2 diabetes.

In total, 30% (339/1130) of participants had ethnicity data, with 18.1% (205/339) from Black, Asian, mixed, or other ethnicities. All participants had IMD data available, with 30.8% (n=348) falling into the lower tertile, 34.3% (n=388) falling into the middle tertile, and 34.9% (n=394) falling into the upper least deprived tertile. A full breakdown of baseline characteristics can be found in Table 1.

**Table 1:** Baseline characteristics of program participants (N=1130).

Characteristic		Values
Age (years), mean (SD)		49.9 (12.0)
Female sex, n (%)		740 (65.4)
BMI (kg/m^2^), mean (SD)		46.3 (31.6)
Weight (kg), mean (SD)		115.7 (21.7)
**IMD^a^ tertile, n (%)**		1130 (100)
	1-3	348 (30.8)
	4-6	388 (34.3)
	7-10	394 (34.9)
**Ethnicity, n (%)**		339 (30)
	Black, Asian, mixed, or others	205 (18.1)
	White	127 (11.2)
	Missing or prefer not to say	798 (70.6)
Presence of type 2 diabetes, n (%)		884 (78.2)

^a^IMD: index of multiple deprivation.

### Weight Change

Of the 1130 participants, 297 (26.2%) recorded weight readings at 1 year from baseline, and 281 (24.9%) recorded weight readings at 2 years from baseline. At the 1-year follow-up, the mean weight loss for those with recorded weights was 10.7 kg (SD 12.3 kg; *P*<.001), equating to a mean percentage weight loss of 9.1% (SD 9.6%; *P*<.001) from baseline. A total of 191 (17%) participants had ≥5% weight loss from baseline, while 107 (9.5%) participants had ≥10% weight loss (Table 2).

**Table 2:** Weight loss outcomes at the 1- and 2-year follow-ups for all participants with recorded weights, all with baseline observation carried forward, and complete cases only.

			At 1-year follow-up	At 2-year follow-up
**All with weight recorded, n (%)**			297 (26.2)	281 (24.9)
	Weight loss (kg), mean (SD)	10.7 (12.3)^a^		13.8 (14.2)^a^
	Weight loss from baseline (%), mean (SD)	9.1 (9.6)^a^		11.8 (10.9)^a^
	≥5% Weight loss from baseline, n (%)	191 (17)		204 (18.1)
	≥10% Weight loss from baseline, n (%)	107 (9.5)		130 (11.5)
**Baseline observation carried forward, n (%)**			1130 (100)	1130 (100)
	Weight loss (kg), mean (SD)	2.8 (7.8)^a^		3.4 (9.2)^a^
	Weight change from baseline (%), mean (SD)	2.4 (6.4)^a^		2.8 (7.3)^a^
	≥5% Weight loss from baseline, n (%)	191 (17)		197 (17.4)
	≥10% Weight loss from baseline, n (%)	107 (9.5)		127 (11.2)
**Complete cases,^b^ n (%)**			207 (18.3)	207 (18.3)
	Weight loss (kg), mean (SD)	10.1 (12.3)^a^		14.7 (14.0)^a^
	Weight change from baseline (%), mean (SD)	9.1 (9.6)^a^		12.5 (10.8)^a^
	≥5% Weight loss from baseline, n (%)	131 (11.6)		156 (13.8)
	≥10% Weight loss from baseline, n (%)	73 (6.5)		105 (9.3)

^a^*P*<.001.

^b^The complete case analyses included participants who had weight readings at both the 1- and 2-year follow-ups.

**Figure 2:**
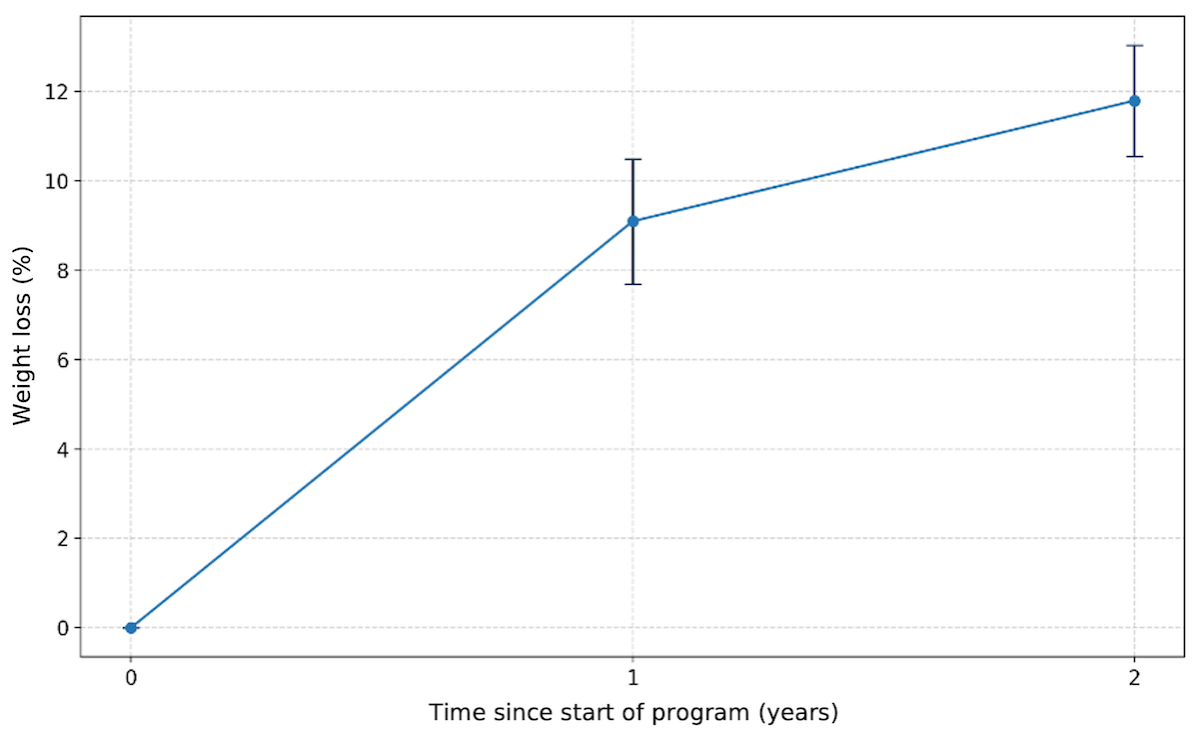
Mean weight loss (%) after 1 year and 2 years. Error bars represent 95% CIs.

The 2-year data also indicated a significant mean weight loss of 13.8 kg (SD 14.2 kg; *P*<.001), which translated to a mean weight loss of 11.8% (SD 10.9%; *P*<.001) from baseline (Figure 2). A total of 204 (18.1%) participants had ≥5% weight loss from baseline, and 130 (11.5%) participants had ≥10% weight loss.

Applying the BOCF method to account for participants who did not record weight readings, the mean weight loss at 1 year was 2.8 kg (SD 7.8 kg; *P*<.001), and at 2 years, it was 3.4 kg (SD 9.2 kg; *P*<.001).

Among completers, those who recorded weights at both 1 year and 2 years, the mean weight loss was 10.1 kg (SD 12.3 kg; *P*<.001) at 1 year and 14.7 kg (SD 14.0 kg; *P*<.001) at 2 years.

### Association Between Baseline Characteristics and Weight Loss

There was no evidence that weight loss at 1 year differed by ethnicity (Black, Asian, mixed, or others vs White) or type 2 diabetes diagnosis. Similarly, at 2 years, there was no evidence that weight loss differed by ethnicity (Black, Asian, mixed, or others vs White) or type 2 diabetes diagnosis, as shown in Table 3.

**Table 3:** Association between baseline participant characteristics and weight loss in kilograms at 1 year and 2 years.

Baseline characteristic		Weight loss from baseline to 1 year^a^		Weight loss from baseline to 2 years^a^	
		β (95% CI)	*P* value	β (95% CI)	*P* value
**Ethnicity (reference=Black, Asian, mixed, or other ethnicities)**					
	White	.77 (–3.8 to 5.3)	.74	.68 (–4.4 to 5.7)	.79
	Prefer not to say	–3.79 (–17.9 to 10.3)	.60	–12.78 (–39.5 to 14.0)	.35
**IMD^b^ tertile (reference=1-3)**					
	4-6	–1.58 (–4.8 to 1.67)	.34	.40 (–3.4 to 4.2)	.84
	7-10	–1.60 (–4.9 to 1.7)	.34	1.46 (–2.5 to 5.4)	.47
**Type 2 diabetes (reference=no)**					
	Yes	1.05 (–2.4 to 4.5)	.55	.24 (–3.8 to 4.3)	.91

^a^All models were adjusted for baseline weight. Separate analyses were run for each baseline characteristic.

^b^IMD: index of multiple deprivation.

### Association Between Engagement and Weight Loss

The mean number of engagements in month 1 was 325 (SD 351.2), rising to 447 (SD 494.7) in month 2, before falling to 313 (SD 313.2) in month 3. There was no evidence that engagement during the “core” phase of the active intervention was associated with weight loss at either 1 year (β=.0007; 95% CI –0.0056 to 0.0071; *P*=.82) or 2 years (β=.0055; 95% CI –0.0026 to 0.0136; *P*=.18). These models were adjusted for baseline weight.

## Discussion

### Principal Results

In this study, we explored the effectiveness of Second Nature’s 12-month DWMI to support adults with obesity, with or without type 2 diabetes, outside of hospital settings to help expand SWMSs for NHS patients. Furthermore, we aimed to contribute to the real-world evidence base on DWMIs and longer-term outcomes of such interventions. Participants demonstrated a statistically significant mean weight loss of 10.7 (SD 12.3) kg, equating to a mean percentage weight loss of 9.1% (SD 9.6%), at 1 year and 13.8 (SD 14.2) kg, which translated to a mean weight loss of 11.8% (SD 10.9%), at 2 years. When analyzed using BOCF, we found a statistically significant mean weight loss of 3.4 (SD 9.2) kg and a mean weight change of 2.8% (SD 7.3%) at 2 years. Weight loss did not significantly differ by ethnicity, IMDs, type 2 diabetes status, or engagement in the program. Overall, these results suggest that Second Nature’s DWMI has the potential to be an effective and equitable DWMI for a diverse NHS patient population living with obesity and comorbid type 2 diabetes and therefore support increased access to SWMS in the NHS.

### Limitations

There were notable limitations within our study. Due to the retrospective, real-world nature of this study, there was no control group, which means the findings must be interpreted carefully. However, a similar study of a commercial DWMI with a larger sample size also found that users lost a significant amount of weight using this type of program [42]. Due to the observational nature of the study, a significant number of participants did not submit weight readings within the specified data collection period, despite regular reminders and encouragement from health coaches. Capturing long-term, real-world data for DWMIs is challenging. Additionally, one-to-one support from health coaches ceased after 3 months of the total program period, which likely contributed to difficulties in capturing longer-term weight data. For the weight and engagement data collected, a self-selection bias is possible, as those participants who weighed themselves more frequently may have been more motivated and engaged and therefore experienced more weight loss.

Participants were referred to Second Nature from tier 2 weight management pathways or as part of routine type 2 diabetes care and not from a SWMS. Nevertheless, patients with obesity are eligible for treatment within SWMS in the NHS at BMI ≥35 kg/m^2^. The average BMI of participants in this study was 46.3 (SD 31.6) kg/m^2^; therefore, many participants would be eligible to access a SWMS. Furthermore, while this program was not initially developed to be a specific “tier 3” program, a distinguishing feature of tier 3 services is an MDT approach. In this study, we worked effectively with the patients’ primary care teams using such an approach, reflecting a similar protocol to existing tier 3 services. Similarly, while we did not have input from an existing tier 3 service, the program was developed by an MDT from Second Nature that consisted of medical doctors, psychologists, dietitians, nutritionists, and behavioral scientists. As such, this study was able to assess the *potential* of a DWMI to support existing SWMS in the NHS.

Due to the retrospective and real-world nature of this analysis, it was not possible to extract and analyze other relevant data such as medication usage, side effects, clinical outcomes (eg, hemoglobin A_1c_, blood pressure, and lipid profile), and psychological and quality of life-related outcomes. Further research is needed to determine the impact of our program on these health outcomes and wider economic impact. Finally, the data used for this study were collected by employees of Second Nature and were not checked by an independent party or NHS organization.

### Comparison With Prior Work

The effectiveness of Second Nature’s DWMI has previously been explored in self-paying consumers and patients with type 2 diabetes; however, these studies included populations with lower average baseline BMIs of 33.7 and 35.9 kg/m^2^, measured shorter-term outcomes at 6 and 12 months [28,43]. This study builds on this earlier work by exploring longer-term outcomes with a population similar to that seen in SWMS [32]. Importantly, an observational study, which assessed the uptake of a commercial DWMI among patients awaiting their first appointment with a SWMS, similarly found their app to be feasible [44]. This study similarly provides preliminary evidence that DWMIs may be a viable way to expand NHS SWMS [19-21]. Remotely delivered interventions have the potential to increase access to treatment for people with busy schedules, limited mobility, and those living in remote areas.

Previous research has found that DWMIs typically require a high amount of personal agency to be effective, given that making such changes to health behaviors requires time, resources, and education [30,31]. Consequently, such interventions risk exacerbating health inequalities and may be inequitable [30,31,45]. In this study, there was no evidence that weight loss differed by ethnicity, IMD, or type 2 diabetes status at follow-up. Similarly, we did not find an association between engagement in the first 12 weeks and weight loss at follow-up. A recent systematic review of 13 studies investigated differences in the uptake of, engagement with, and effectiveness of mobile interventions for weight-related by age, gender, race and ethnicity, and socioeconomic status [46]. Given the limited number of studies and inconsistent findings, the authors stated that current evidence of the presence of a digital divide in mobile interventions targeting weight-related behaviors is inconclusive [46]. However, further research, such as a randomized controlled trial with a larger sample size, is warranted to support the findings of this study.

To continue building the evidence base on DWMIs, it would also be beneficial to explore the impact of the collaboration of a DWMI and MDT including dietitians, doctors, psychologists, and exercise specialists on outcomes for people living with obesity and related conditions with the view to increase safety and accountability and optimize treatment outcomes. Additionally, an evaluation of the integration of pharmacotherapeutic interventions embedded in DWMIs for SWMSs is also needed.

### Conclusions

This study suggests that Second Nature’s DWMI has the potential to support people living with obesity and type 2 diabetes remotely to achieve clinically significant and sustained weight loss at 2 years from starting an intervention. DWMIs could help to expand existing SWMS outside of hospital settings to increase access to treatment and reduce pressure on hospitals. Further research is needed to compare such interventions to standard care as well as assess the integration of DWMIs with multidisciplinary clinical teams and pharmacotherapy to support this study’s findings.

## Abbreviations

BOCF: baseline weight observation carried forward
DWMI: digital weight management intervention
IMD: index of multiple deprivation
MDT: multidisciplinary team
NHS: National Health Service
SWMS: specialist weight management service

## References

[1] (2023). Health Survey for England, 2021. NHS Digital.

[2] (2022). National Survey for Wales headline results: April 2021 to March 2022. Welsh Government.

[3] (2023). Obesity in Scotland: prevalence, causes and impact. Obesity Action Scotland.

[4] (2023). Health Survey Northern Ireland 2019/20: first results. Department of Health.

[5] Wharton S, Lau DCW, Vallis M, Sharma AM, Biertho L, Campbell-Scherer D, Adamo K, Alberga A, Bell R, Boulé N, Boyling E, Brown J, Calam B, Clarke C, Crowshoe L, Divalentino D, Forhan M, Freedhoff Y, Gagner M, Glazer S, Grand C, Green M, Hahn M, Hawa R, Henderson R, Hong D, Hung P, Janssen I, Jacklin K, Johnson-Stoklossa C, Kemp A, Kirk S, Kuk J, Langlois MF, Lear S, McInnes A, Macklin D, Naji L, Manjoo P, Morin MP, Nerenberg K, Patton I, Pedersen S, Pereira L, Piccinini-Vallis H, Poddar M, Poirier P, Prud'homme D, Salas XR, Rueda-Clausen C, Russell-Mayhew S, Shiau J, Sherifali D, Sievenpiper J, Sockalingam S, Taylor V, Toth E, Twells L, Tytus R, Walji S, Walker L, Wicklum S (2020). Obesity in adults: a clinical practice guideline. CMAJ.

[6] Whitlock G, Lewington S, Sherliker P, Clarke R, Emberson J, Halsey J, Qizilbash N, Collins R, Peto R (2009). Body-mass index and cause-specific mortality in 900 000 adults: collaborative analyses of 57 prospective studies. Lancet.

[7] Abdullah A, Peeters A, de Courten M, Stoelwinder J (2010). The magnitude of association between overweight and obesity and the risk of diabetes: a meta-analysis of prospective cohort studies. Diabetes Res Clin Pract.

[8] (2022). Estimating the full costs of obesity. Frontier Economics.

[9] (2013). Clinical commissioning policy: complex and specialised obesity surgery. NHS Commissioning Board.

[10] Alkharaiji M, Anyanwagu U, Donnelly R, Idris I (2019). Tier 3 specialist weight management service and pre-bariatric multicomponent weight management programmes for adults with obesity living in the UK: a systematic review. Endocrinol Diabetes Metab.

[11] Hazlehurst JM, Logue J, Parretti HM, Abbott S, Brown A, Pournaras DJ, Tahrani AA (2020). Developing integrated clinical pathways for the management of clinically severe adult obesity: a critique of NHS England policy. Curr Obes Rep.

[12] Coulton V, Dodhia S, Ells L, Blackshaw J, Tedstone A (2015). National mapping of weight management services: provision of tier 2 and tier 3 services in England. Public Health England.

[13] Watkins R, Swancutt D, Alexander M, Moghadam S, Perry S, Dean S, Sheaff R, Pinkney J, Tarrant M, Lloyd J (2023). A qualitative exploration of patient and staff experiences of the receipt and delivery of specialist weight management services in the UK. Patient.

[14] (2023). New drugs pilot to tackle obesity and cut NHS waiting lists. UK Government.

[15] (2023). Semaglutide for managing overweight and obesity. National Institute for Health and Care Excellence.

[16] (2023). Digital technologies for delivering specialist weight-management services to manage weight-management medicine: early value assessment. National Institute for Health and Care Excellence.

[17] Brown TJ, O'Malley C, Blackshaw J, Coulton V, Tedstone A, Summerbell C, Ells LJ (2017). Exploring the evidence base for tier 3 weight management interventions for adults: a systematic review. Clin Obes.

[18] Huntriss R, Haines M, Jones L, Mulligan D (2021). A service evaluation exploring the effectiveness of a locally commissioned tier 3 weight management programme offering face-to-face, telephone and digital dietetic support. Clin Obes.

[19] Sorgente A, Pietrabissa G, Manzoni GM, Re F, Simpson S, Perona S, Rossi A, Cattivelli R, Innamorati M, Jackson JB, Castelnuovo G (2017). Web-based interventions for weight loss or weight loss maintenance in overweight and obese people: a systematic review of systematic reviews. J Med Internet Res.

[20] Schippers M, Adam PCG, Smolenski DJ, Wong HTH, de Wit JBF (2017). A meta-analysis of overall effects of weight loss interventions delivered via mobile phones and effect size differences according to delivery mode, personal contact, and intervention intensity and duration. Obes Rev.

[21] Beleigoli AM, Andrade AQ, Cançado AG, Paulo MN, Diniz MDFH, Ribeiro AL (2019). Web-based digital health interventions for weight loss and lifestyle habit changes in overweight and obese adults: systematic review and meta-analysis. J Med Internet Res.

[22] Ross KM, Carpenter CA, Arroyo KM, Shankar MN, Yi F, Qiu P, Anthony L, Ruiz J, Perri MG (2022). Impact of transition from face-to-face to telehealth on behavioral obesity treatment during the COVID-19 pandemic. Obesity (Silver Spring).

[23] Fischer M, Weimann T, Oberänder N, Schupitza L, Hösel J, Weimann A (2022). Remote treatment successfully delivers a usual care weight loss and lifestyle intervention in adults with morbid obesity. Ann Nutr Metab.

[24] Rothberg AE, Marriott DJ, Miller NM, Herman WH (2023). Retention and weight outcomes after transitioning an intensive behavioral weight management program from an in-person to a virtual format. Obes Sci Pract.

[25] Hanson P, Summers C, Panesar A, Oduro-Donkor D, Lange M, Menon V, Barber TM (2021). Low Carb Program health app within a hospital-based obesity setting: observational service evaluation. JMIR Form Res.

[26] Hartmann-Boyce J, Johns DJ, Jebb SA, Summerbell C, Aveyard P, Behavioural Weight Management Review Group (2014). Behavioural weight management programmes for adults assessed by trials conducted in everyday contexts: systematic review and meta-analysis. Obes Rev.

[27] Second Nature.

[28] Idris I, Hampton J, Moncrieff F, Whitman M (2020). Effectiveness of a digital lifestyle change program in obese and type 2 diabetes populations: service evaluation of real-world data. JMIR Diabetes.

[29] Ross JAD, Barron E, McGough B, Valabhji J, Daff K, Irwin J, Henley WE, Murray E (2022). Uptake and impact of the English National Health Service digital diabetes prevention programme: observational study. BMJ Open Diabetes Res Care.

[30] Adams J, Mytton O, White M, Monsivais P (2016). Why are some population interventions for diet and obesity more equitable and effective than others? The role of individual agency. PLoS Med.

[31] Backholer K, Beauchamp A, Ball K, Turrell G, Martin J, Woods J, Peeters A (2014). A framework for evaluating the impact of obesity prevention strategies on socioeconomic inequalities in weight. Am J Public Health.

[32] (2023). Obesity: identification, assessment and management. Clinical guideline [CG189]. National Institute for Health and Care Excellence.

[33] (2014). Weight management: lifestyle services for overweight or obese adults. Public health guideline [PH53]. National Institute for Health and Care Excellence.

[34] (2015). Preventing excess weight gain. NICE guideline [NG7]. National Institute for Health and Care Excellence.

[35] (2012). Type 2 diabetes: prevention in people at high risk. Public health guideline [PH38]. National Institute for Health and Care Excellence.

[36] (2014). Behaviour change: individual approaches. Public health guideline [PH49]. National Institute for Health and Care Excellence.

[37] (2017). Obesity: working with local communities. Public health guideline [PH42]. National Institute for Health and Care Excellence.

[38] (2016). NHS Diabetes prevention programme: an opportunity to partner with the behavioural insight team to improve outcomes. Public Health England and NHS England Behavioural Insights Team.

[39] Michie S, van Stralen MM, West R (2011). The behaviour change wheel: a new method for characterising and designing behaviour change interventions. Implement Sci.

[40] (2019). English indices of deprivation 2019. Ministry of Housing, Communities & Local Government.

[41] Holzapfel C, Cresswell L, Ahern AL, Fuller NR, Eberhard M, Stoll J, Mander AP, Jebb SA, Caterson ID, Hauner H (2014). The challenge of a 2-year follow-up after intervention for weight loss in primary care. Int J Obes (Lond).

[42] Serrano KJ, Yu M, Coa KI, Collins LM, Atienza AA (2016). Mining health app data to find more and less successful weight loss subgroups. J Med Internet Res.

[43] Kar P, Goward C, Whitman M, Davies M, Willner T, Shaw K (2020). Engagement and effectiveness of digitally enabled behavioural change support for people living with type 2 diabetes. Pract Diabetes.

[44] Hanson P, Summers C, Panesar A, Liarakos AL, Oduro-Donkor D, Oshodi DW, Hailston L, Randeva H, Menon V, de la Fosse M, Kaura A, Shuttlewood E, Loveder M, Poole D, Barber TM (2023). Implementation of a digital health tool for patients awaiting input from a specialist weight management team: observational study. JMIR Hum Factors.

[45] Birch JM, Jones RA, Mueller J, McDonald MD, Richards R, Kelly MP, Griffin SJ, Ahern AL (2022). A systematic review of inequalities in the uptake of, adherence to, and effectiveness of behavioral weight management interventions in adults. Obes Rev.

[46] Szinay D, Forbes CC, Busse H, DeSmet A, Smit ES, König LM (2023). Is the uptake, engagement, and effectiveness of exclusively mobile interventions for the promotion of weight-related behaviors equal for all? A systematic review. Obes Rev.

